# Editorial: Interplay of inflammation and schizophrenia: pathophysiology and therapeutic opportunities

**DOI:** 10.3389/fpsyt.2026.1965875

**Published:** 2026-09-02

**Authors:** Declan P. McKernan, Ernest Marek Tyburski

**Affiliations:** 1 School of Pharmacy and Medical Sciences, University of Galway, Galway, Ireland; 2 Institute for Health Discovery and Innovation, University of Galway, Galway, Ireland; 3 Department of Health Psychology, Pomeranian Medical University in Szczecin, Szczecin, Poland

**Keywords:** biomarkers, immune dysregulation, inflammation, precision psychiatry, psychosis, schizophrenia

## 1 Introduction

Alterations in peripheral inflammatory cytokines are well reported in schizophrenia but are incompletely understood. Inflammatory profiles in schizophrenia are heterogeneous: some alterations persist across acute and chronic stages, whereas others appear stage-specific, underscoring the need for analyses to incorporate illness stage (1). Earlier publications showed an association of immune dysregulation with genetic variants, environmental exposures, and antipsychotic use (2, 3). More recent research has moved beyond isolated cytokines by integrating cell types, neuroimaging, cerebrospinal fluid analyses, and integrated data-driven approaches to explain biological heterogeneity (4). Multivariate blood–brain profiles in early psychosis further support the feasibility of biologically informed clinical stratification (5). However, causal pathways and clinical utility remain uncertain. This Research Topic adds to the literature on biological roles of inflammatory cytokines and their clinical relevance in schizophrenia with clinical studies, systematic reviews, case reports and animal models.

## 2 Inflammation: cumulative burden and clinical heterogeneity

The concept of allostatic load in schizophrenia is investigated by Madaria et al. Here, they broaden the interpretation of inflammation in schizophrenia by incorporating cytokine levels with neuroendocrine, cardiovascular and metabolic markers using a meta-analysis. The authors report that higher allostatic load in first-episode psychosis and chronic schizophrenia-spectrum disorders suggests that immune alterations are part of a cumulative metabolic, neuroendocrine, and cardiovascular dysregulation. Comparability remains limited across the studies investigated due to variation in the composition and calculation of allostatic-load indices. Another factor considered in this Research Topic was that of childhood trauma. Laffely et al. showed childhood trauma and unfavorable parental relationships were associated with elevated lipid and prolactin levels during the first year of antipsychotic treatment in first-episode psychosis. Despite including methodologically heterogeneous studies, Ingabire et al. identified predominantly positive associations between peripheral immune markers and negative-symptom severity in their systematic review, with interleukin-1β (IL-1β) and tumor necrosis factor-alpha (TNF-α) showing the most reproducible associations. Conversely, Heil et al. found no evidence that longitudinal changes in peripheral inflammatory indices tracked changes in psychopathology. Elsewhere, Szwajca et al. reported associations of complement components C3/C4 with symptom severity on the PANSS but these did not survive false-discovery-rate correction. Together, these findings favor multiple immune-related profiles rather than a single inflammatory phenotype of schizophrenia.

## 3 Peripheral immune signals alter brain and gut physiology

How peripheral immune signals influence brain physiology remains unresolved. Amin et al. investigated how serum TNF-α levels influence hippocampal, prefrontal, and ventricular volume. Patients were reported to have lower TNF-α levels and larger lateral ventricles than controls. A weak inverse association between TNF-α and prefrontal cortex volume was also observed but only within the schizophrenia group. In a rat maternal immune-activation model, Zhu et al. reported reduced hippocampal volume, impaired white-matter integrity, and neurochemical abnormalities, some of which were partially modulated by risperidone. In an exploration of the microbiota–gut–brain axis, Xu et al. reported distinct immunoglobulin G and M (IgG/IgM)-coated gut microbiota populations in fecal samples from patients with schizophrenia. They also reported that fecal microbiota transplantation (FMT) from these patients into mice led to intestinal inflammation. These animal studies support biologically plausible links between peripheral immunity, the gut, and the brain but do not establish causal pathways; therefore, the results cannot be directly extrapolated to humans.

## 4 Immune stratification and prediction of psychosis trajectories

The clinical utility of immune stratification is not merely whether an immune marker differentiates psychosis from health, but whether it improves prognostic classification and treatment selection. The PEPAMARKER protocol aims to do this. Its prospective, multicenter first-episode psychosis cohort integrates linguistic, clinical, and inflammatory data and purportedly predicts affective versus non-affective trajectories at 24 months (Terrisse et al.). This design is important because a single inflammatory measure is unlikely to achieve sufficient prognostic accuracy. The heterogeneity summarized by Ingabire et al. and the negative longitudinal findings of Heil et al. reinforce the need to integrate immune measures with illness stage, symptom dimensions, cognition, metabolic status, medication exposure, and neurobiological data. Longitudinal, multimodal stratification is therefore a more realistic objective than a stand-alone diagnostic test.

## 5 Therapeutic opportunities: promise, precision, and safety

If immune stratification in schizophrenia is to become clinically relevant, it needs to demonstrate utility in predicting therapeutic efficacy. In a case report, Murray et al. reported that a five-week ketogenic diet in a woman with treatment-resistant schizophrenia and metabolic syndrome coincided with improvements in insulin resistance, fasting insulin, C-reactive protein, and pseudoparkinsonism, without meaningful improvement in global psychopathology. Because sustained ketosis was achieved only during the final five days, this report is hypothesis-generating rather than evidence of efficacy. Additionally, Zhu et al. suggest that risperidone may modulate structural, microstructural, and metabolic correlates in the hippocampus in a rat model of maternal immune activation. These data require replication in larger experimental samples and eventually in a human cohort. Translation must also prioritize safety. In a case report, Lv et al. documented a rare, bilateral hand-restricted drug-induced rash temporally associated with blonanserin, emphasizing the importance of prompt recognition and individualized pharmacovigilance. Case reports can identify safety issues at an individual level but cannot substitute for controlled trials in well-characterized subgroups.

## 6 Conclusion: from inflammation to precision psychiatry

Across this Research Topic, progress depends on greater comparability, reproducibility, and clinical interpretability. Immune-marker panels and composite biological indices require standardization. Repeated longitudinal assessments are essential because a single measure may reflect a transient state rather than a stable biological dimension. Studies should systematically account for antipsychotic exposure, smoking, body mass index and metabolic syndrome, diet, physical activity, sleep, infection, and substance use. Priority should be given to integrating immune measures with neuroimaging, microbiota, cognition, symptom phenomenology, and functioning. The field must progress from documenting statistical associations to establishing clinical validity, prognostic value, and therapeutic utility. The eleven contributions in this Research Topic strengthen the conclusion that immune and inflammatory processes are relevant to a subset of individuals with schizophrenia, while also demonstrating marked heterogeneity in their biological expression and clinical implications. The goal for the field should be the identification of immune-relevant mechanisms and patient subgroups in which such information is clinically actionable.
